# Electrically Controlled Neurochemical Delivery from Microelectrodes for Focal and Transient Modulation of Cellular Behavior

**DOI:** 10.3390/bios11090348

**Published:** 2021-09-20

**Authors:** Chao Tan, Neetu Kushwah, Xinyan Tracy Cui

**Affiliations:** 1 Department of Bioengineering, University of Pittsburgh, Pittsburgh, PA 15261, USA; cht117@pitt.edu (C.T.); nek36@pitt.edu (N.K.); 2 Center for Neural Basis of Cognition, Pittsburgh, PA 15213, USA; 3 McGowan Institute for Regenerative Medicine, Pittsburgh, PA 15219, USA

**Keywords:** drug delivery, cell culture, biosensing, fluorescence imaging

## Abstract

Electrically controlled drug delivery of neurochemicals and biomolecules from conducting polymer microelectrode coatings hold great potentials in dissecting neural circuit or treating neurological disorders with high spatial and temporal resolution. The direct doping of a drug into a conducting polymer often results in low loading capacity, and the type of molecule that can be released is limited. Poly(3,4-ethylenedioxythiophene) (PEDOT) doped with sulfonated silica nanoparticles (SNP) has been developed as a more versatile platform for drug delivery. In this work, we demonstrate that neurochemicals with different surface charge, e.g., glutamate (GLU), gamma-Aminobutyric acid (GABA), dopamine (DA), 6,7-Dinitroquinoxaline- 2,3-dione (DNQX) and bicuculline, can be, respectively, incorporated into the SNP and electrically triggered to release repeatedly. The drug loaded SNPs were incorporated in PEDOT via electrochemical deposition on platinum microelectrodes. After PEDOT/SNP(drug) coating, the charge storage capacity (CSC) increased 10-fold to 55 ± 3 mC/cm^2^, and the impedance at 1 kHz was also reduced approximately 6-fold. With the aid of a porous SNP, the loading capacity and number of releases of GLU was increased >4-fold and 66-fold, respectively, in comparison to the direct doping of PEDOT with GLU (PEDOT/GLU). The focal release of GLU and GABA from a PEDOT/SNP (drug) coated microelectrode were tested in cultured neurons using Ca imaging. The change in fluo-4 fluorescence intensity after electrically triggered GLU (+6.7 ± 2.9%) or GABA (−6.8 ± 1.6%) release indicated the successful modulation of neural activities by neurotransmitter release. In addition to activating neural activities, glutamate can also act on endothelial cells to stimulate nitric oxide (NO) release. A dual functional device with two adjacent sensing and releasing electrodes was constructed and we tested this mechanism in endothelial cell cultures. In endothelial cells, approximately 7.6 ± 0.6 nM NO was detected in the vicinity of the NO sensor within 6.2 ± 0.5 s of GLU release. The rise time of NO signal, T_0–100_, was 14.5 ± 2.2 s. In summary, our work has demonstrated (1) a platform that is capable of loading and releasing drugs with different charges; (2) proof of concept demonstrations of how focal release of drugs can be used as a pharmacological manipulation to study neural circuitry or NO’s effect on endothelial cells.

## 1. Introduction

Miniaturized microelectrodes are favorable in neuroscience due to the reduced invasiveness, increased spatial resolution and selectivity. Microelectrodes have been extensively used in electrophysiology [1], electrical microstimulations [2], in vivo imaging [3], and chemical sensors [4]. Microelectrodes can also be used to study neurochemical interplay and neural circuitry dynamics when coupled to drug delivery tools. For example, sensory-evoked neural network activity in rat primary somatosensory cortex was studied by delivering GLU receptor antagonist, 6,7-Dinitroquinoxaline- 2,3-dione (DNQX) [5]. Neurochemical signals have been extensively reported when excitative or inhibitive drugs were injected to region of interest [6,7,8]. These studies rely on pharmacological perturbation to localized cell populations. Microcapillary tubes or microfluidic injection tools are often employed in such studies. However, these methods require additional hardware and are subject to drug leakage or system integration complexity.

Conducting polymer coatings on microelectrodes offer an attractive alternative. Conducting polymers have been widely used for improving neural electrode recording and stimulation performances by increasing effective surface area, reducing impedance, improving bioelectronic signal, and increasing charge injection limit [9,10,11,12]. Conducting polymers can be electropolymerized on any conductive surfaces with the polymer backbone to become positively charged, attracting negatively charged dopants in the film. Dopant molecules may be released upon the application of a sufficient negative current. Due to this unique behavior, conducting polymers provides an electrically controlled mechanism for on demand drug delivery. Various types of dopants have been incorporated into conducting polymers, including anti-inflammatory species such as dexamethasone [5], α-amino-3-hydroxy-5-methyl-4-isoxazolepropionic acid (AMPA) receptor antagonist 6-cyano-7-nitroquinoxaline-2,3-dione (CNQX) and DNQX [13], or neurotransmitters such as dopamine [14]. Conducting polymer drug delivery has been used in multi-modal systems. In a recent study, Xu et al. developed a close-loop design for wound management where they used a voltage-controlled system to deliver anti-bacterial cefazolin to treat infected wounds on the backs of rats, and meanwhile monitored key parameters, such as temperature, PH, and uric acid concentration of the infected areas [15].

One of the limitations of the aforementioned drug delivery is that the drug needs to be negatively charged. Another challenge is the quantity of drug loading, which needs to be increased to meet the requirement of the in vitro and in vivo application. As such, porous nanomaterials, such as graphene oxide nanosheets [16], carbon nanotubes [17], etc., have been utilized as drug carriers to improve the quantity of drug loading and tune the release rate. Recently, silica-based sulfonated nanoparticles (SNPs) have been used as conducting polymer dopants, which serve as drug reservoirs for electrically controlled drug delivery. SNPs not only increase drug load and the range of drug choices, but can also protect electroactive drugs such as melatonin from losing their redox efficacy during electropolymerization [12].

In this work, the loading and releasing of a variety of drugs (positive, negative, neutral charge) from PEDOT/SNP coated microelectrodes were tested. The incorporation of SNP not only increased the loading capacity, but also improved the lifetime and stability of the coated drug layer. The key excitatory neurotransmitter glutamate and inhibitory transmitter GABA were released in primary neuronal culture to demonstrate the ability to focally modulate neuronal activities. In endothelial cells, we were able to activate the endothelial nitric oxide synthase (eNOS) through GLU delivery and detect NO production using a NO sensor adjacent to the drug delivery electrode. Taken together, the technology reported in this study (1) serve as a useful pharmacological manipulation method to modulate the cellular behavior at high spatial and temporal resolution; (2) can easily be combined with other analytical modalities to achieve multimodal interrogation of the biological system and on demand drug delivery for variety of therapeutic applications in the future.

## 2. Materials and Methods

### 2.1. The Materials and Characterization

All chemicals were purchased from Sigma-Aldrich (St. Louis, MO, USA) and used as received unless otherwise stated. GLU oxidase (GLUOx) was purchased from Cosmo Bio (Carlsbad, CA, USA). PFA-coated 127 µm-diameter Platinum microwires were obtained from A-M Systems (Sequim, WA, USA). Molecular Probes Fluo-4 calcium kit was purchased from Thermo Fisher Scientific (Waltham, MA, USA). Electrochemical characterizations were performed using Autolab PGSTAT128N (Metrohm, Utrecht, The Netherlands). Fluorescence spectroscopy measurement were performed on Molecular Devices SpectraMax i3x (San Jose, CA, USA) using a microplate reader. Fluorescence images were taken from a Leica DMIRB inverted microscope equipped with a Prime 95B sCMOS camera from Teledyne Photometrics (Tucson, AZ, USA).

### 2.2. Drug Loading and Polymerization

The synthesis of porous SNP can be found in our previous work [12]. Briefly, tetraethyl orthosilicate and mercaptopropyl trimethoxysilane were used to synthesize silica nanoparticles, and the inclusion of trimethylammonium produced a porous structure. Hydrogen peroxide was then used to oxidize thiol groups to sulfonate, and the resulting negatively charged SNPs can serve as a dopant for conducting polymer. For drug loading, 10 mg SNP was first mixed with 700 mg GLU, 1300 mg GABA, or 100 mg DA in 1 mL DI water, or 5 mg of DNQX or bicuculline in 200 µL DI water. The solution was sonicated for 30 min and then centrifuged, collected and dried under vacuum overnight. The dried drug loaded SNPs were then washed with water and centrifuged 5 times to wash away the free drug. Finally, 15 mM EDOT in 2 mL DI water was mixed with 5 mg drug loaded SNP and sonicated for 20 min. Electropolymerization was carried out using Ag/AgCl as the reference electrode and a platinum (Pt) foil as the counter electrode. Then, 40 nA was applied to the 127 µm-diameter platinum microwire electrode for 1000 s. PEDOT/SNP(drug) coated Pt microwires were then gently dipped in DI water for 30 s and stored dry before use.

### 2.3. Drug Release and Quantifications

**Scheme 1:**
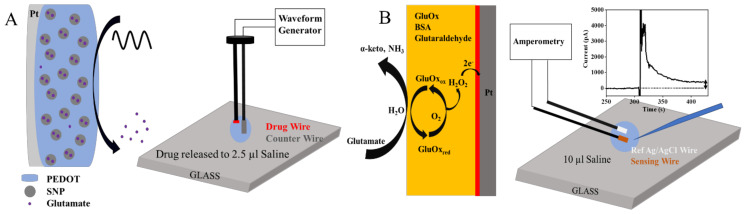
(**A**) Schematic drawing of GLU (drug) release from conducting polymer film by applying a sine wave voltage. (**B**) The released GLU was then detected by GLU biosensor through the detection of enzymatic byproduct, H_2_O_2_. Amperometry: +0.7 V vs. Ag/AgCl.

For drug release, a sine waveform (2 Hz, 0 ± 0.5 V, burst mode) was applied to the Pt/PEDOT/SNP(drug) microwire using an Agilent 33120A waveform generator and a 1 cm-length Pt microwire was used as the counter electrode. The tip of drug microwire and counter electrodes were closely placed on a small drop of 2.5 µL PBS solution on a glass slide sitting on the top of a Petri dish filled with DI water to maintain the humidity ***(***Scheme 1A). After each set of stimuli (e.g., 60, 120, 180, 240, 300 stimuli, or 30, 60, 90, 120, 150 s), the solution was collected and stored in microcentrifuge tubes. Another drop of fresh 2.5 µL PBS was then applied for the next release stimulus. The quantification of released GLU used a GLU oxidase-based amperometric sensor, where the enzymatic byproduct hydrogen peroxide was oxidized at +0.7 V and measured with amperometry (Scheme 1B). The quantification of GABA used a modified GABA fluorescent kit where the collected solution was mixed with the GABA kit and incubated at 37 °C. The resulting fluorescent product resorufin is measured based on the fluorescence (550 nm excitation and 590 nm emission) using a microplate reader. DA was quantified with amperometry using a Pt/Nafion sensor with a bias voltage of +0.3 V. DNQX and bicuculline were quantified with peak absorbance at 285 nm using a microplate reader.

### 2.4. Microelectrode Preparation

The drug electrode used the cross-sectional area of a 127 µm-diameter platinum microwire as substrate. As for the NO or GLU sensor, a 1 mm length tip of a platinum wire was exposed and used. Microwires were sonicated in IPA and then CV cycled in sulfuric acid. To engineer an NO sensor, meta-phenylenediamine (mPD) was coated onto Pt by CV cycling ((0.2, 0.8 V), 50 mV/s, 50 cycles) the microwire in N_2_ purged 5 mM mPD solution. Pt/mPD microsensors with a biased voltage of +0.9 V vs. Ag/AgCl were used as working electrodes for NO detection. GLU biosensor was coated with an additional layer of enzyme (0.4 U/µL GLUOx in bovine serum albumin and glutaraldehyde solution) and operated at 37 °C using amperometry at +0.7 V vs. Ag/AgCl.

### 2.5. Cell Cultures

Primary neurons were isolated from E18 rat fetuses. E18 pregnant Sprague Dawley rats were euthanized under CO_2_, followed by decapitation. Rat embryos were removed carefully and sterilized with 70% ethanol solution, followed by separating the brain cortex. Meninges were removed gently, and brain cortical tissues were transferred to ice cold HBSS solution. Cortical tissues were then incubated and homogenized in 0.25% trypsin/EDTA for 15 min at 37 °C and then washed 2–3 times with ice cold HBSS and mechanically dispersed by repetitive trituration with a 1 mL fine tip. Then, 10 μL aliquots of cell suspension were added to 10 μL of trypan blue solution before counting by hemocytometer. Only cells considered viable were counted. Neurons were plated at a density of 25,000 cells/well in 48 well plates in B27/N2/GlutaMax/PenStrep supplemented Neurobasal Media and grown for 7–14 days, with half of the media changed every third day. To increase the validity, cultures were repeated 3 times.

The isolation of primary rat brain endothelial cells was performed as per the previously well-described protocols with minor modifications [18,19]. Briefly, 5 week-old Sprague Dawley rats were sacrificed by cervical dislocation and the cerebral hemispheres were carefully separated and placed into ice cold PBS buffer. Meninges weredetached by careful rolling of the brains on a sterile blotting paper. After eliminating the meninges from the forebrains, the brain tissues were minced in a homogenizer and later digested with an enzymatic solution (collagenase and DNAse) for 1 hr at 37 °C in an orbital shaker at 180 rpm. To separate the myelin, the suspension was then mixed with 20% (*W*/*V*) BSA/DMEM. The sediment was then digested by mixed enzymatic solution again; during digestion, a density gradient percoll solution was set up using an ultracentrifuge at 3000× *g* for 1 h at 4 °C, and then the digested solution was separated at 700× *g* for 10 min at 4 °C. The cells were clearly visible as a cloud in the interphase. The interphase was taken with a long sterile needle and cells were centrifuged again at 1000× *g* for 10 min at 4 °C. The pellet was resuspended in DMEM/F12 supplemented with 20% bovine serum, 20 μg/mL basic fibroblast growth factor (bFGF), 100 μg/mL heparin, 100 μg/mL gentamicin, and 2.5 mM HEPES with 4 mg/mL puromycin. The resuspended pellet was transferred to collagen type IV and fibronectin precoated culture plates. Cells passaged 2–3 times were used in this study. Endothelial cells were maintained at 37 °C during the electrochemical measurement using a water bath circulation system.

### 2.6. Calcium Imaging in Neuronal Culture

Calcium imaging was used to evaluate the effect of electrically released GLU and GABA in cultured neurons. Fluo-4 calcium imaging kit was used for the detection of calcium influx during drug release. Calcium-bound Fluo-4 dye has an excitation of 494 nm and emission of 506 nm. In addition, 20 mM glucose in 2 mL live cell imaging solution was added to the well to support the cell health in the longer term. A single drug wire (Pt/PEDOT/SNP(GLU) or Pt/PEDOT/SNP(GABA)) was inserted perpendicularly into the Petri dish cultured with live neurons. With the aid of a Leica DMIRB microscope, the drug wire was positioned immediately adjacent to or in contact with the cell layer. Electrical stimulations (2 Hz, 0 ± 0.5 V, burst mode) were then applied, and the stimulation duration was set to 5 s. The fluorescence images were acquired at 5 frame/sec for 30 s, which covers the 5 s before, 5 s onset, and 20 s after stimulation. The exposure time was set to 10 ms. The calcium signal of individual neurons was analyzed using ImageJ. The baseline fluorescence intensity F_0_ was obtained by averaging the signal before stimulation. ΔF is the fluorescence intensity after F_0_ subtraction.

### 2.7. NO Sensing in Endothelial Culture

The NO sensing experiment used two microwire electrodes, one for drug release and one for NO sensing. The tip of the two microwires (~1 cm) were bundled together using a heat-shrink tube. The tips of the two wires were then positioned parallel to horizontal direction and lowered to the bottom of well plate that was cultured with endothelial cells using a micromanipulator. The well plate was placed in a water-bath system with the temperature maintained at 37 °C. The GLU drug wire and a platinum counter wire electrode were connected to the two ends of the waveform generator to trigger GLU release. The NO sensing wire and an Ag/AgCl reference/counter were connected to the Autolab potentiostat running chronoamperometry with the biased voltage of +0.9 V to monitor NO upon the release of GLU to neighboring endothelial cells.

## 3. Results and Discussion

### 3.1. SNP Enhanced Surface Behavior and Drug Release

**Figure 1:**
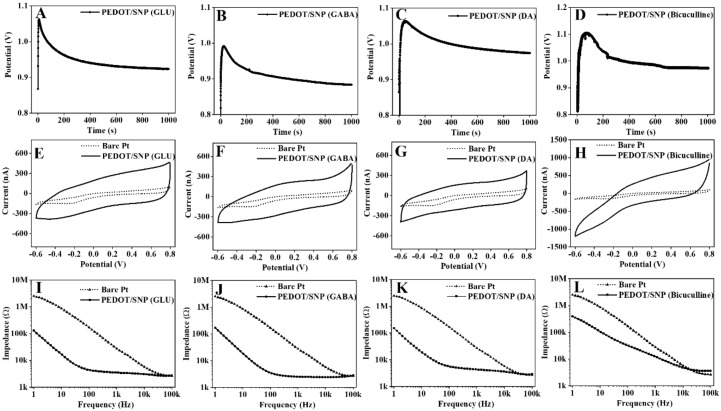
Chronopotentiometry coating (**A**–**D**), CV scan (**E**–**H**), and EIS (**I**–**L**) of drug loaded conducting polymer films on Pt surface. These films include PEDOT/SNP (GLU), PEDOT/SNP (GABA), PEDOT/SNP (DA) and PEDOT/SNP (Bicuculline). The applied charge density for the coating deposition was 320 mC/cm^2^.

PEDOT/SNP(drug) coating was synthesized using chronopotentiometry with the applied charge density of 320 mC/cm^2^ (40 nA, 1000 s). The chronopotentiometry data for PEDOT/SNP(GLU), PEDOT/SNP(GABA), PEDOT/SNP(DA), and PEDOT/SNP (bicuculline) were shown in Figure 1A–D. After an initial charging surge, a gradual decrease in electrode potential is observed for all coatings, indicating a continuous decrease in electrode impedance as the PEDOT/SNP coating grows, a typical characteristic of the conducting polymer. Cyclic voltammetry (–0.6 to +0.8 V, 100 mV/s) and electrochemical impedance spectroscopy (1 to 100 kHz) were used to characterize the platinum electrode before and after all PEDOT/SNP(drug) coatings (Figure 1E–H and I–L).

A general increase in charge storage capacity (CSC) and decrease in impedance magnitude in frequencies between 1 and 10k Hz were observed for all coatings. Specifically, for PEDOT/SNP(GLU), a 10-fold increase in CSC (from 5.8 ± 0.4 to 60.8 ± 12.9 mC/cm^2^) and an 18-fold (2590 ± 126.2 kΩ to 139.3 ± 7.0 kΩ at 1 Hz) or 6-fold (29.7 ± 1.5 kΩ to 5.2 ± 1.0 kΩ to at 1 kHz) decrease in impedance were observed after the coating. The CSC and impedance of all coatings are shown in Table 1. The same characterization was performed for PEDOT/SNP(DNQX), and same trend was observed (Figure S1A–C). These results all suggested a dramatic improvement in electrochemical behavior after coating, regardless of the drug types examined.

**Figure 2:**
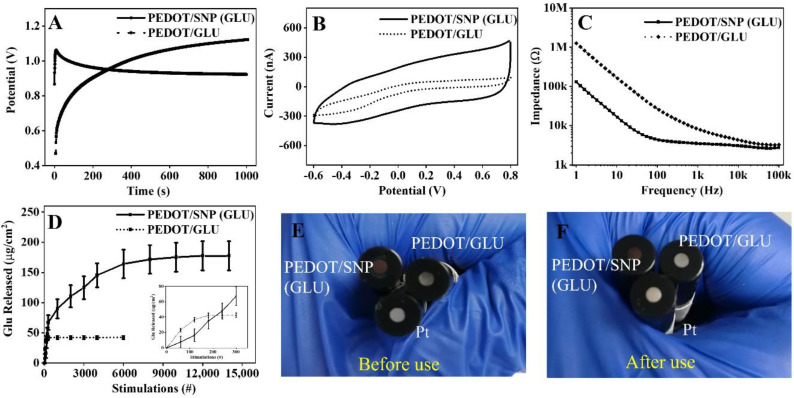
(**A**–**D**) Chronopotentiometry coating, CV scan, EIS and release profile comparison between PEDOT/SNP(GLU) and PEDOT/GLU. Insect in panel D shows release data from the first 300 stimulations for better visualization. (**E**,**F**) Optical micrographs of Pt, PEDOT/SNP(GLU), and PEDOT/GLU coated Pt macroelectrodes (2 mm diameter) before and after 1000 stimulation pulses.

It is noteworthy that negatively charged drugs can be directly used as the dopant to the cationic conducting polymer, PEDOT. Thus, the polymerized film without SNP was also studied for comparison. We electropolymerized Pt surface with PEDOT/GLU using the same coating charge density, and the coating curve of PEDOT/GLU was different from PEDOT/SNP (GLU), and the electrode potential increased with the growth of film, indicating a more resistive film formation that prevents further growth of the film (Figure 2A). Further analysis of CV and EIS on the PEDOT/GLU film showed a much smaller CSC and higher impedances than PEDOT/SNP(GLU) (Figure 2B,C).

To quantify glutamate release, we constructed a glutamate oxidase-based glutamate sensor following a previously published protocol [20]. The sensor was calibrated against glutamate solutions of different concentrations, and a linear current response (I after and before addition of solution) was observed between 0–20 µM (Figure S2A,B). The drug release solutions were then applied to the sensor to determine the released GLU amount (example shown in Figure S2C). GLU release from PEDOT/GLU film only worked for 90 s of release stimulation (three collections), and no detectable signal was observed thereafter. The total amount of GLU released from PEDOT/GLU was 42.2 ± 3.0 µg/cm^2^ (Figure 2D and Figure S2C). On the other hand, with a pre-loading of GLU to SNP, we were able to continuously trigger the release of GLU from PEDOT/SNP (GLU) film for 12,000 stimulations (6000 s, 2 Hz), and the total amount of GLU released was 177.7 ± 24.1 µg/cm^2^. Compared to the drug film without SNP, this is a >4-fold increase in drug releasing capacity and 66-fold increase in lifetime. It should be noted that, to minimize the passive release of drug that was loosely incorporated in the conducting polymer layer, we washed the SNP before electropolymerization to dispose of most of the free GLU in the supernatant. Experiments were done to confirm that there was no detectable diffusion of GLU from the washed coating, and electrically stimulating PEDOT/SNP without a loaded drug does not result in signal detection from the sensor (Figure S3).

To better understand the differences between PEDOT/GLU and PEDOT/SNP (GLU), we coated macroelectrodes using these two types of films, respectively. Optical images of the two surfaces have clearly demonstrated that, under the same coating condition, the PEDOT/SNP (GLU) layer was dark black and fully covering the macroelectrode surface, while the PEDOT/GLU was only slightly darker than a bare Pt electrode (Figure 2E), indicating the PEDOT/GLU layer was much thinner than the PEDOT/SNP (GLU) layer. After stimulation for 1000 pulses, the PEDOT/GLU coating appeared to have degraded/detached, while the PEDOT/SNP (GLU) remain unchanged (Figure 2F). The superiority of PEDOT/SNP (GLU) is due to the porous structure of the nanoparticle that helps trap more GLU, as well as the better doping capability of SNP, resulting in greater conductivity, a high surface area, and adherent coating. Together, our data have suggested an improved electrochemical behavior, drug loading and releasing capability, as well as stimulation stability from the PEDOT/SNP(GLU) film.

**Figure 3:**
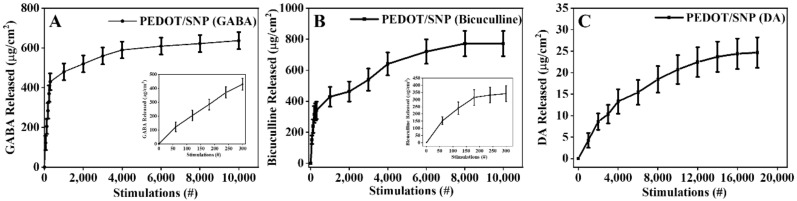
(**A**–**C**) Release profile of drug loaded microwires; the films are PEDOT/SNP (GABA), PEDOT/SNP (Bicuculline), and PEDOT/SNP (DA). Insect in panel A and B shows data from first 300 stimulations only.

In addition to negatively charged GLU, we also characterized several other drug candidates that are neurotransmitters or modulators of neural activities. These include the zwitterionic GABA, positively charged bicuculline and dopamine, and negatively charged DNQX. This involved releasing 637 ± 42.8 µg/cm^2^ of GABA in 10,000 stimulations; 771 ± 81 µg/cm^2^ of bicuculline were released in 8000 stimulations; 24.7 ± 3.6 µg/cm^2^ of DA were released in 18,000 stimulations (Figure 3); and 329 ± 38 µg/cm^2^ DNQX were released in 6000 stimulations.

### 3.2. Focal Release of Neurotransmitters Induced Localized Neuronal Excitation and Inhibition

**Figure 4:**
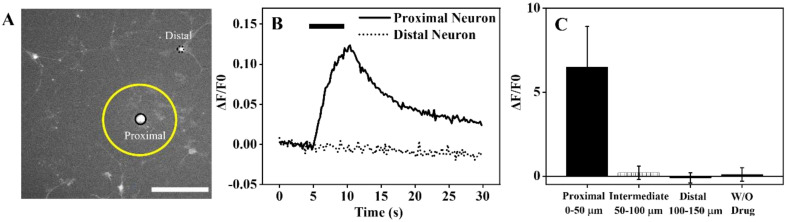
(**A**) Fluorescent calcium imaging of neuron activities upon electrically stimulated GLU released from microwire coated with PEDOT/SNP(GLU). Yellow circle indicates the position of drug releasing site on microwire, black solid circle labels a proximal neuron and dash circle labels a distal neuron, scale bar is 100 µm. (**B**) Fluorescence intensity changes of the two example neurons in (**A**) upon electrically stimulated GLU release, horizontal line indicates the stimulation duration. Image capture rate was 200 ms/frame for 30s. (**C**) Maximum intensity change of neurons averaged from three separate cultures at different distances, as well as the electrical control group, where empty PEDOT/SNP electrodes were stimulated. No Ca^2+^ change was observed when electrodes were 50 µm away or without GLU loading.

We evaluated the efficiency and resolution of our drug delivery system in neuronal culture, using Ca^2+^ imaging to indicate the neural activities. With the incorporation of drug releasing from microelectrode and imaging of cultured neurons under a high resolution microscope, it is possible to achieve single cell analysis. In our experiment, we delivered either GLU or GABA to cultured neurons and observed significant changes in calcium signal of neurons in local area. For GLU delivery, the fluorescence intensity of neurons near the drug releasing microwire increased upon the triggering of stimulation. An example image is shown in Figure 4A,B; the fluorescent intensity of a neuron near the releasing electrode (proximal) increased immediately after the stimulus onset at 5 s and decreased shortly after the stimulus was turned off at 10 s. On the other hand, a distal neuron that was 150 µm away did not show any fluorescence change (Figure 4B). The peak ΔF/F_0_ from neurons of three separate cultures was averaged and is shown in Figure 4C. The neurons within 50 µm of the GLU releasing electrode showed a +6.7 ± 2.9% increase in Ca^2+^ signals upon stimulated GLU release, while the neurons from 50–100 and 100–150 µm had negligible change, indicating the high spatial resolution of the release technology. To validate that the observed Ca^2+^ activities were from the released chemical GLU rather than the physical effect of electrical stimulations, we repeated the experiment but used Pt wire coated with PEDOT/SNP without a drug and found no change after the identical stimulation (N = 5) (Figure 4C) without drug group. Thus, our results demonstrated the capability of our electrical triggered GLU delivery technology in transiently activating neurons in a highly localized manner.

**Figure 5:**
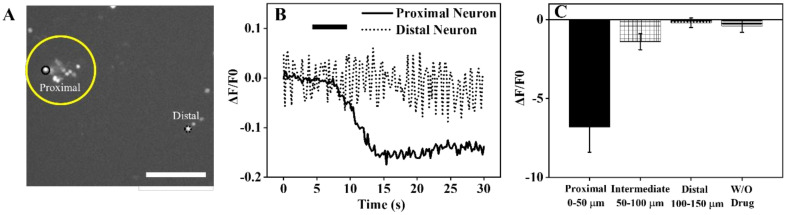
(**A**) Fluorescent calcium imaging of neuron activities upon electrically stimulated GABA released from microwire coated with PEDOT/SNP(GABA). Yellow circle indicates the position of drug releasing site on microwire, black solid circle labels a proximal neuron and dash circle labels a distal neuron, scale bar is 100 µm. (**B**) Fluorescence intensity changes of the two example neurons shown in (**A**), upon electrically stimulated GABA release, horizontal line indicates the stimulation duration. Image capture rate was 200 ms/frame for 30 s. (**C**) Maximum intensity decrease in neurons averaged from three separate cultures at different distances, as well as the electrical control group where empty PEDOT/SNP electrodes were stimulated. Highest Ca^2+^ activity reduction is found in neurons with the first 50 µm of the releasing site, smaller response in the intermediate zone of 50–100 µm, and no Ca^2+^ change was observed between 100–150 µm away or without GABA loading.

A similar study was performed on neurons using microwires coated with PEDOT/SNP(GABA). As shown in the example image in Figure 5A,B, the Ca^2+^ signal of the proximal neuron decreased approximately 2.4 s after the stimulation onset, while the distal neuron was not affected. On average, neurons within 50 µm of the GABA releasing site showed a 6.8 ± 1.6% maximum decrease in Ca^2+^ signals, while neurons in the 50–100 µm zone showed a small reduction of −1.4 ± 0.5%. Distal neurons (100–150 µm) did not change in fluorescence intensity (Figure 5C). Again, stimulating non-drug PEDOT/SNP electrodes in the same cultures did not elicit a Ca^2+^ signal change.

These data demonstrate the proof of concept of using the electrically controlled neurochemical release from microelectrode to induce highly localized excitation and inhibition, which can be a powerful tool to study neuronal network dynamics.

### 3.3. GLU Stimulated NO Release in Cultured Endothelial Cells

GLU is not only an important neurotransmitter that can cause excitatory response from neurons, but also involved in the dynamics of other cellular functions. For example, GLU may bind to endothelial nitric oxide synthase (eNOS) and activate the production of NO, which is an important small molecule involved in vascular dilation, neurovascular coupling with demonstrated anti-inflammatory and neuroprotective effects. In a previous study, the release of NO was reported when rat hippocampus was stimulated with 5 mM GLU in brain slices [21]. However, bath incubation of GLU offers low temporal and spatial resolution. Here, we demonstrate our ability to stimulate NO release using focal delivery of GLU from the PEDOT/SNP(GLU) coated microwires.

For the detection of NO upon GLU delivery in cultured endothelial cells, the drug wire was modified with PEDOT/SNP(GLU) only on its tip, and the 1 mm length Pt wire was used for NO detection (Figure S7A,B). In order to detect NO, the Pt wire was coated with a screening layer of mPD to increase the selectivity over possible interferents [22]. The cyclic voltammogram ((0.2, 0.8 V), 50 mV/s, 50 cycles) of coating mPD on Pt surface was obtained and the oxidation current decreased as the cycles continued, which was due to the increased coverage of non-conductive mPD (Figure S5A). Due to the resistivity of the mPD coating, some redox peaks belonging to Pt were diminished after mPD was coated (Figure S5B,C). For sensor calibration, a NO solution was prepared by dissolving diethylenetriamine/nitric oxide (DETA/NO) adduct in argon purged 1X PBS solution. The oxidation peak of NO was at +0.85 V vs. Ag/AgCl (Figure S6A), which agrees with previous studies [22]. The sensitivity and selectivity of a Pt/mPD wire sensor were tested. Size-exclusive mPD layer has effectively impeded the majority of signal from ascorbic acid (AA), a main interferent in the brain (Figure S6B,C). Our NO calibration curve showed a linear fitting from 10 nM–1 µM with an NO sensitivity of 46.9 pA/nM, R^2^ = 0.988 (Figure S6D,E). The calculated selectivity of NO:AA and NO:NO_2_^−^ were 10,679 ± 1833 and 586 ± 33, respectively. Thus, our sensor is highly selective to NO. It is noteworthy that the DETA/NO adduct had a 100:1 concentration ratio when releasing NO, and this ratio was also confirmed by using NO produced from chemical synthesis method where potassium iodide and potassium nitrite in sulfuric acid solution were used (Figure S6F).

**Figure 6:**
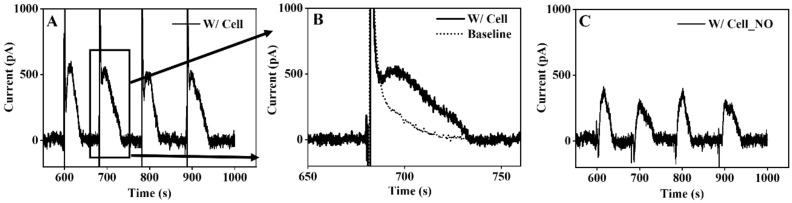
(**A**) Amperometry recording in cultured endothelial cells with electrical stimulations applied. (**B**) An enlarged current response curve showing the increased current after the stimulation artifact. (**C**) Baseline subtracted signal. The applied potential is +0.9 V vs. Ag/AgCl.

After a stable baseline was obtained from the sensing electrode (>10 min), electrical stimulations (1 s, 2 Hz) were applied to the drug wire repeatedly, and the resulting current responses in the neighboring NO sensing electrode were recorded (Figure 6). As expected, electrical artifacts of stimulation together with NO current increase were observed after each stimulation (Figure 6A,B). After subtracting the baseline, the net current response, mostly from the NO, is shown in Figure 6C. We repeated the experiment in endothelial cells with the biased potential set to +0.6 V or in media-only (without cells) at +0.9 V; both showed no signals following the electrical artifact, which indicates that the measured current signal was from a chemical that has an oxidation potential at +0.9 V, and this chemical is produced by the endothelial cells (Figure S8). To sum up, this study demonstrated robust NO generation when GLU was released to endothelial cells from PEDOT/SNP (GLU) microelectrodes. The response time (from end of stimulation to 0% of spike) of NO generation here was approximately 6.2 ± 0.5 s. The peak concentration of NO observed from our experiment was 7.6 ± 0.6 nM with a rise time T_0–100_ (from 0% to 100% of spike) of 14.5 ± 2.2 s and recovery time of 31 ± 4.2 s (from peak to baseline). Compared to the bath application, our drug delivery technology provides a much faster and localized effect and can be used to study the kinetics of different signaling events at cellular resolution. This dual function device design may also allow on demand delivery of therapeutic glutamate when the NO level is detected to be below a threshold.

## 4. Conclusions

Unlike many studies that have released only one type of molecule and used traditional electrical trigger methods, such as cyclic voltammetry or amperometry [5,12,13,14], we successfully loaded a variety of neurochemicals with negative, positive, and zwitterionic charges (GLU, GABA, DA, DNQX, and bicuculline) into PEDOT/SNP deposited onto microelectrode surfaces and used a novel sine wave voltage pulse stimulation. Passive release of the drug was often observed and reported from previous work [15]; however, in this study, drug molecules in our system can be released in a controllable fashion for thousands of times. The excitatory and inhibitory neurotransmitter, GLU and GABA, were electrically delivered from microelectrodes to cultured neurons and caused a corresponding increase or decrease (~ ±6.8%) in the calcium activities of neurons local to the releasing electrode. A GLU drug releasing electrode and an NO sensing electrode were then used together to achieve simultaneous delivery of GLU and NO sensing in endothelial cells. These proof-of-concept experiments demonstrate the great potential for using the PEDOT/SNP drug delivery system for focal manipulation of cellular activities at previously unattainable spatial and temporal resolutions. Additionally, combining the focal drug delivery with sensing and recording electrode will further enable the study of molecular interplay as well as the development of feedback controlled drug delivery systems. The limitation of our system is that our drug delivery can only effectively affect cells within several 10 s of microns distance. This is useful for applications that require very fine spatial control but would be insufficient when a larger volume of tissue needs to be affected. Furthermore, we do not have a direct refill mechanism. Other drug delivery systems from microelectrode devices, such as electrophoretic ion pumps, may provide longer lasting and therapeutic effects [23,24]. For clinical applications, future development of our technology will consider incorporation of external reservoirs and delivery channels.

## Figures and Tables

**Table 1:** CSC and impedance of all drugs loaded conducting polymer coatings.

Materials	CSC (mC/cm^2^)	Impedance @ 1 Hz (kΩ)	Impedance @ 1 kHz (kΩ)
Pt	5.8 ± 0.4	2590.0 ± 126.2	29.7 ± 1.5
PEDOT/GLU	8.5 ± 0.3	1123.7 ± 79.1	6.8 ± 0.9
PEDOT/SNP (GLU)	60.8 ± 12.9	139.3 ± 7.0	5.2 ± 1.0
PEDOT/SNP (GABA)	36.9 ± 3.8	161.7 ± 12.0	2.3 ± 0.1
PEDOT/SNP (DA)	36.2 ± 2.4	163.7 ± 7.4	4.6 ± 0.4
PEDOT/SNP (Bicuculline)	72.6 ± 4.3	212.7 ± 16.2	12.2 ± 0.2

## Data Availability

Not applicable.

## Supplementary Materials

The following are available online at https://www.mdpi.com/article/10.3390/bios11090348/s1, Figure S1. Electro-polymerization and Characterization of PEDOT/SNP (DNQX). (A) Chronopotentiometry during electropolymerization, (B) cyclic voltammetry before and after PEDOT/SNP (DNQX) coating, (C) electrochemical impedance spectroscopy, and (D) release profile. Figure S2. (A) Calibration of an enzyme-based GLU sensor showing amperometry detection of 0–20 µM GLU, measured at +0.7 V vs. Ag/AgCl. (B) Linear fitting of GLU calibration curve, y = 0.246x, R^2^ = 0.98. C) Amperometric detection of GLU released from a PEDOT/SNP (GLU) or PEDOT/GLU film between 1000 and 2000 stim. Figure S3. (A) Amperometric detection of GLU from a soak (500 s)-release (500 s)-soak (3000 s) three-step to demonstrate there was no obvious leak of GLU from PEDOT/SNP (GLU) film without the electrical trigger. (B) Solution released from a PEDOT/SNP coated Pt wire did not contribute to faradaic current change, +0.7 V vs. Ag/AgCl. Figure S4. (A–D) Calibration curve of GABA sensing (0–20 µM) using a fluorescent kit, DA sensing (0–5 µM) using a platinum/Nafion sensor, DNQX sensing (0–50 µM) and bicuculline sensing (0–100 µM) using absorbance spectroscopy. (E–H) Linear fitting of GABA fluorescence calibration curve, y = 0.0143x, R^2^ = 0.99; linear fitting of DA sensing calibration curve, y = 0.0143x, R^2^ = 0.99; linear fitting of DNQX absorbance calibration curve, y = 0.00067x, R^2^ = 0.992; linear fitting of bicuculline absorbance calibration curve, y = 0.00052x, R^2^ = 0.996. Figure S5. (A) Electropolymerization of mPD on Pt surface, mPD coating process uses CV scan between (0.2, 0.8 V), 50 mV/s, 50 cycles. (B) and (C) indicate that the new surface is covered by mPD and is less conductive. Figure S6. (A) Cyclic voltammogram of Pt/mPD in 200 µM NO in 1X PBS to indicate the oxidation peak of NO. (B) Amperometry recording of a Pt with 200 µM AA to depict a large signal from AA without mPD. (C) Amperometry recording of a Pt/mPD with 250 µM KNO_2_ to show that NO_2_^-^ can be detected. (D) Calibration curve of a Pt/mPD with 200 µM AA, 10 nM–1 µM NO. (E) Linear fitting of NO calibration curve, k = 46.9 pA/nM, R^2^ = 0.988. (F) Calibration curve of a Pt/mPD with 80–320 nM NO produced from chemical synthesis: 2KI + 2KNO_2_ + 2H_2_SO_4_ = I_2_ + 2Na_2_SO_4_ + 2H_2_O + 2NO. Amperometry: +0.9 V vs. Ag/AgCl. Figure S7. (A) SEM picture of cross section area from PEDOT/SNP (GLU) drug microwire. (B) Optical picture of sensing microwire and drug wire placed close in one heat-shrink tube. Figure S8. (A) Amperometry recording in cultured endothelial cells with the applied potential of +0.6 V vs. Ag/AgCl. (B) Amperometry recording in media-only well, and the applied potential is +0.9 V vs. Ag/AgCl.
