# Supplementary material for: Electrically Controlled Neurochemical Delivery from Microelectrodes for Focal and Transient Modulation of Cellular Behavior
Source: Biosensors (Basel). 2021 Sep 20;11(9):348. doi: 10.3390/bios11090348 (PMC8467485; doi:10.3390/bios11090348)
Supplement: Supplementary file 1 [file biosensors-11-00348-s001.zip › biosensors-1370005-supplementary.pdf]

Supplementary Information

Electrically Controlled Neurochemical Delivery From Microelectrodes For Focal and Transient Modulation of Cellular Behavior

Authors:

Chao Tan [1], Neetu Kushwah [2], Xinyan Tracy Cui [1,2,3,*]

[1] Department of Bioengineering, University of Pittsburgh, Pittsburgh, PA 15261, USA

[2] Center for Neural Basis of Cognition, Pittsburgh, PA 15213, USA

[3] McGowan Institute for Regenerative Medicine, Pittsburgh, PA 15219, USA

* Corresponding author. Department of Bioengineering, University of Pittsburgh, 5057 Biomedical Science Tower 3, 3501 Fifth Avenue, Pittsburgh, PA, 15260, USA. E-mail address: xic11@pitt.edu (X.T. Cui).

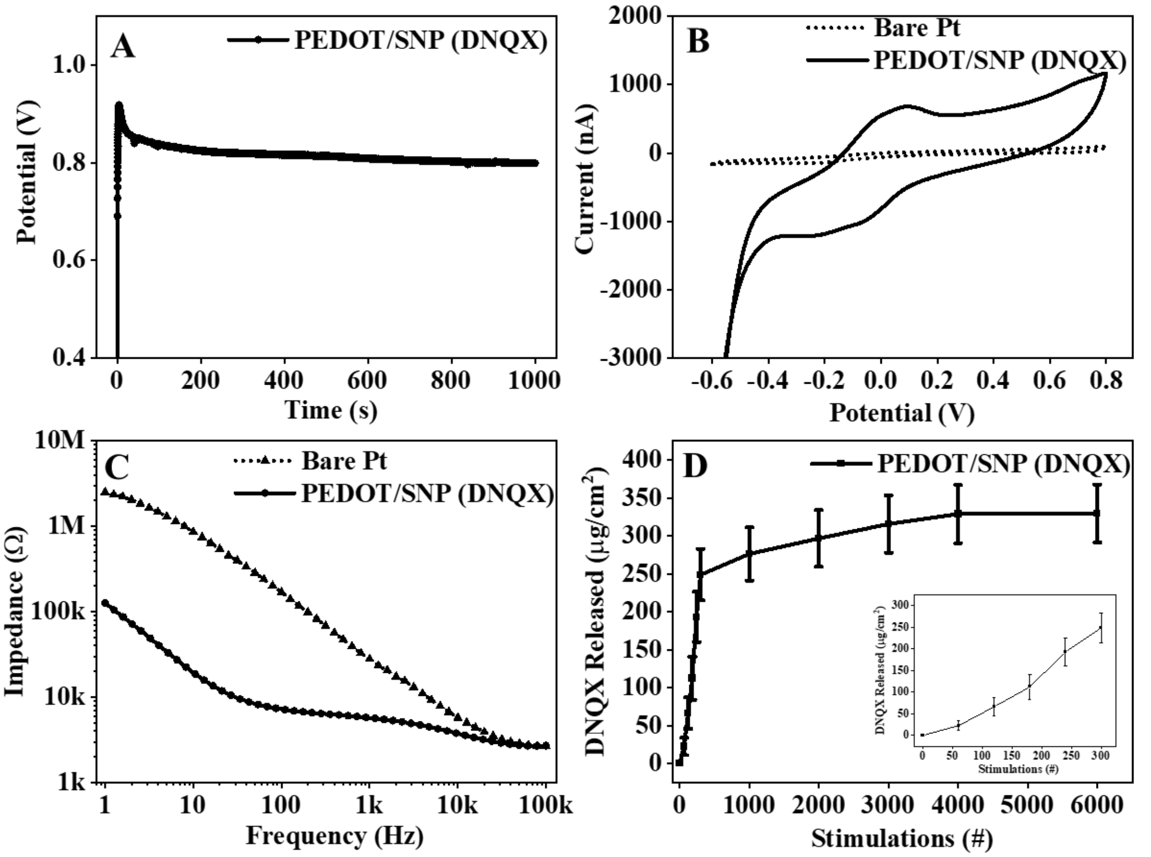

Figure S1. Electro-polymerization and Characterization of PEDOT/SNP (DNQX). A) Chronopotentiometry during electropolymerization, (B) cyclic voltammetry before and after PEDOT/SNP (DNQX) coating, (C) electrochemical impedance spectroscopy, and (D) release profile.

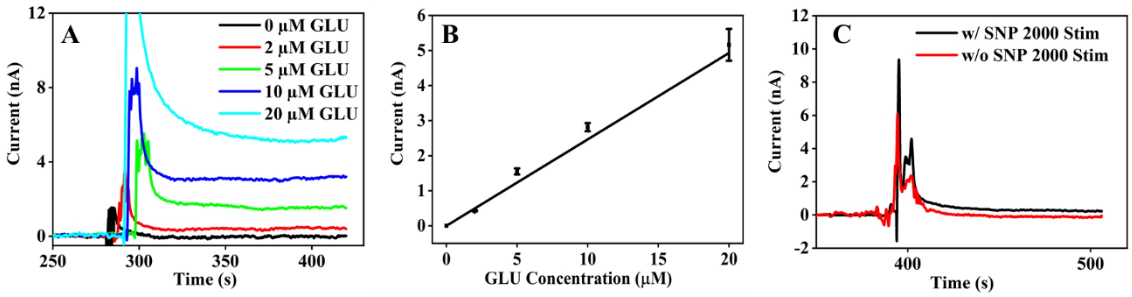

Figure S2. A) Calibration of an enzyme-based GLU sensor showing amperometry detection of 0–20 µM GLU, measured at +0.7 V vs. Ag/AgCl. B) Linear fitting of GLU calibration curve, y = 0.246x, R^2 = 0.98. C) Amperometric detection of GLU released from a PEDOT/SNP (GLU) or PEDOT/GLU film between 1000 and 2000 stim.

GLU biosensor was used for a precise measurement of GLU released from porous SNP. The sensitivity of GLU biosensor was measured from an amperometric calibration experiment with the GLU concentration ranges between 0-20 µM, *Figure S2.A*. GLU signal current was fitted linearly (R^2=0.98) with concentrations as showing in *Figure S2.B* and the GLU sensitivity was determined to be 246 ±21 pA/µM. For drug quantification measurement, the collected GLU samples (2.5 µl) were applied to the surface of GLU biosensor, and amperometry current increase from baseline was used to quantify the GLU released from Pt/PEDOT/SNP (GLU). *Figure S2.C* showed that after 1000-2000, while the GLU release from PEDOT/SNP (GLU) was clearly observed based on the current increase from baseline (after the artifact due to motion of adding sample), there was no GLU in collected drug solution stimulations from PEDOT/GLU.

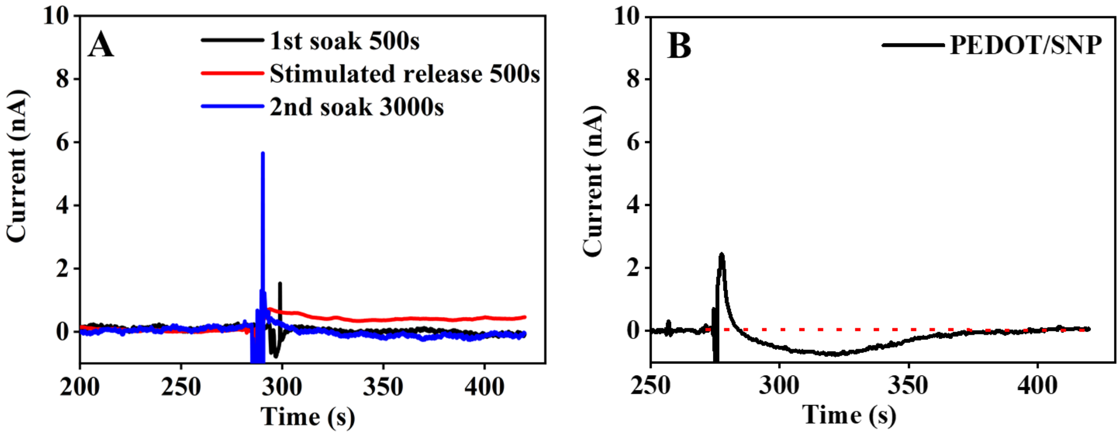

Figure S3. A) Amperometric detection of GLU from a soak (500s)-release (500s)-soak (3000s) three-step to demonstrate there was no obvious leak of GLU from PEDOT/SNP (GLU) film without the electrical trigger. B) Solution released from a PEDOT/SNP coated Pt wire did not contribute to faradaic current change, +0.7 V vs. Ag/AgCl.

This data confirms that 1) our drug delivery system does not passively release detectable amount of drug without the trigger (both before and after stimulation), and 2) the amperometry signal is originated from GLU released, not EDOT monomer or SNP.

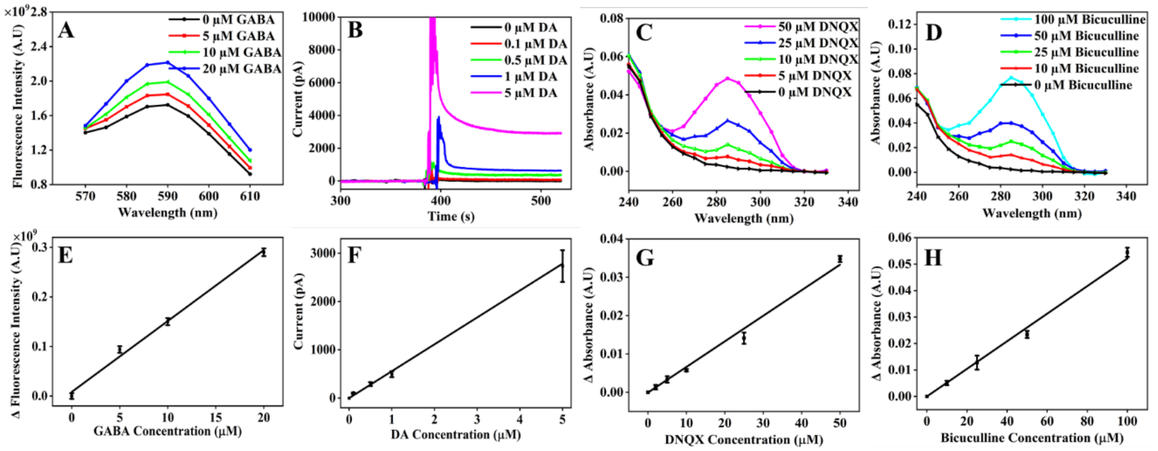

Figure S4. (A–D) Calibration curve of GABA sensing (0–20 μM) using a fluorescent kit, DA sensing (0–5 μM) using a platinum/Nafion sensor, DNQX sensing (0–50 μM) and bicuculline sensing (0–100 μM) using absorbance spectroscopy. (E–H) Linear fitting of GABA fluorescence calibration curve, y = 0.0143x, R^2 = 0.99; linear fitting of DA sensing calibration curve, y = 0.0143x, R^2 = 0.99; linear fitting of DNQX absorbance calibration curve, y = 0.00067x, R^2 = 0.992; linear fitting of bicuculline absorbance calibration curve, y = 0.00052x, R^2 = 0.996.

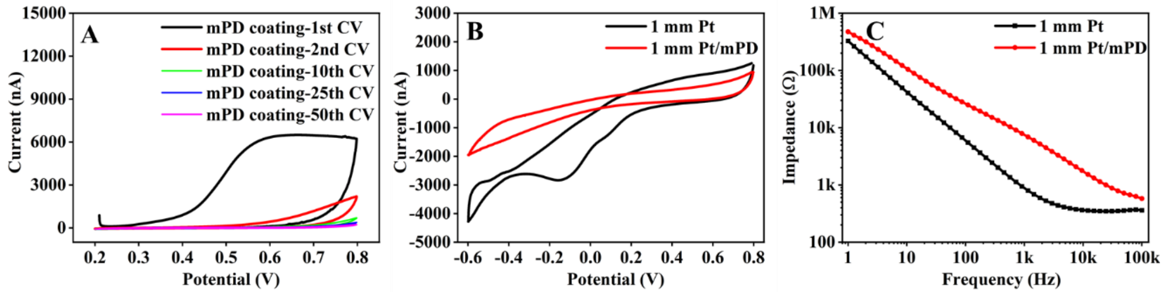

Figure S5. A) Electropolymerization of mPD on Pt surface, mPD coating process uses CV scan between (0.2, 0.8V), 50 mV/s, 50 cycles. B) and C) indicate that the new surface is covered by mPD and is less conductive.

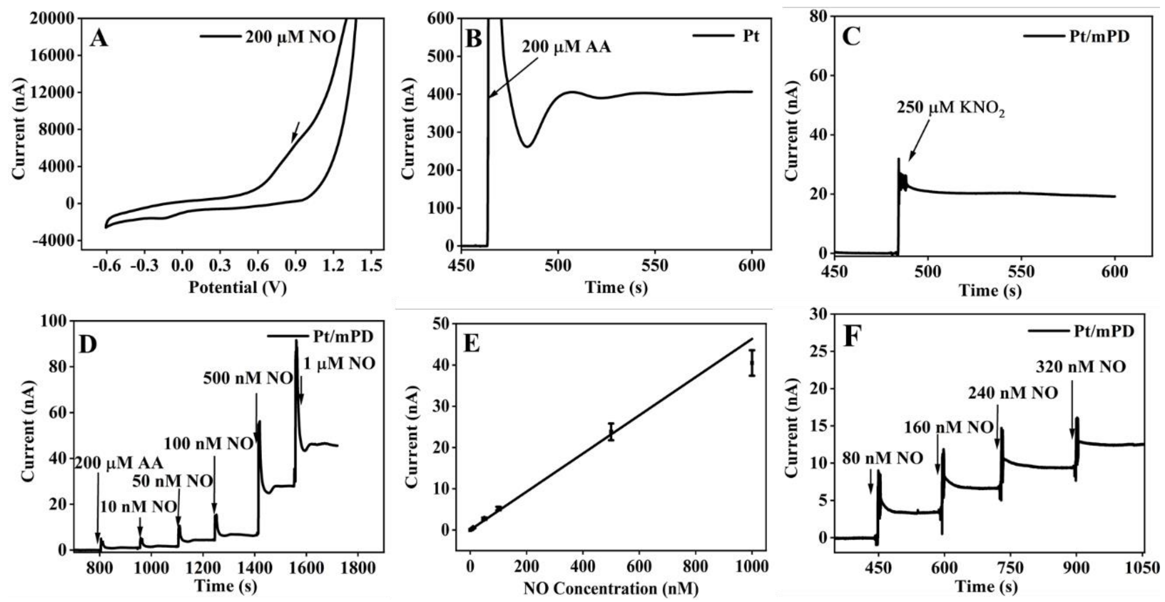

Figure S6. A) Cyclic voltammogram of Pt/mPD in 200 μM NO in 1X PBS to indicate the oxidation peak of NO. B) Amperometry recording of a Pt with 200 μM AA to depict a large signal from AA without mPD. C) Amperometry recording of a Pt/mPD with 250 μM KNO_2 to show that NO_2^- can be detected. D) Calibration curve of a Pt/mPD with 200 μM AA, 10 nM-1 μM NO. E) Linear fitting of NO calibration curve, k = 46.9 pA/nM, R^2 = 0.988. F) Calibration curve of a Pt/mPD with 80–320 nM NO produced from chemical synthesis: $2KI + 2KNO_2 + 2H_2SO_4 = I2 + 2Na_2SO_4 + 2H_2O + 2NO$. Amperometry: +0.9 V vs. Ag/AgCl.

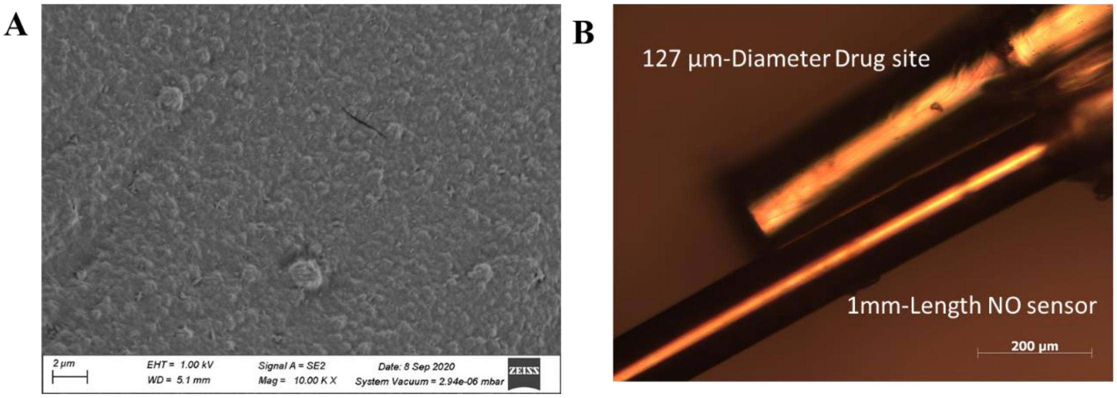

Figure S7. A) SEM picture of cross section area from PEDOT/SNP (GLU) drug microwire. B) Optical picture of sensing microwire and drug wire placed close in one heat-shrink tube.

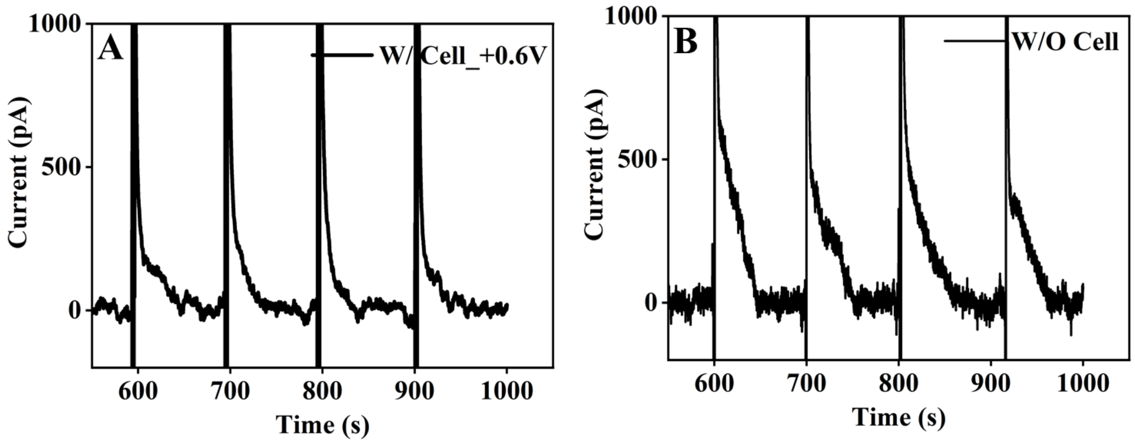

Figure S8. A) Amperometry recording in cultured endothelial cells with the applied potential of +0.6V vs. Ag/AgCl. B) Amperometry recording in media-only well, and the applied potential is +0.9V vs. Ag/AgCl.
